# Sexual Orientation, Gender Identity, and Romantic Relationships in Adolescents and Adults with Autism Spectrum Disorder

**DOI:** 10.1007/s10803-017-3199-9

**Published:** 2017-06-09

**Authors:** J. Dewinter, H. De Graaf, S. Begeer

**Affiliations:** 1 Scientific Centre for Care & Welfare (Tranzo), Tilburg University, Tilburg, The Netherlands; 2 GGzE Centre for Child & Adolescent Psychiatry, PO Box 909 (DP1104), 5600 AX Eindhoven, The Netherlands; 3 Rutgers, Utrecht, The Netherlands; 4 Faculty of Behavioural and Movement Sciences, VU Amsterdam, Amsterdam, The Netherlands

**Keywords:** Autism spectrum disorder, Sexual orientation, Romantic relationships, Gender identity, Adolescents, Adults

## Abstract

This study compared sexual orientation and romantic relationship experience in a large sample of adolescents and adults with autism spectrum disorder (ASD) (n = 675) and general population peers (n = 8064). Gender identity was explored in the ASD group in relation to assigned gender at birth. Compared to general population peers, more people with ASD, especially women, reported sexual attraction to both same- and opposite-sex partners. About half of the participants with ASD was in a relationship (heterosexual in most cases) and most of them lived with their partner. A notable number of autistic participants, again more women than men, reported gender non-conforming feelings. Attention to gender identity and sexual diversity in education and clinical work with people with ASD is advised.

## Introduction

Experience with solo and partnered sexuality and with romantic relationships is common for the majority of adolescents and adults with autism spectrum disorder (American Psychiatric Association 2013) without an intellectual disability (Byers and Nichols 2014; Byers et al. 2012; Dewinter et al. 2016, 2015; Strunz et al. 2017). Evidence for the view of sexuality as a normative part of adolescent development and adult functioning in ASD is relatively recent, and contrasts with earlier views that a majority of adolescents and adults with ASD is asexual, or that sexuality is a problematic issue for them (Bertilsdotter Rosqvist 2014; Dewinter et al. 2013; Kellaher 2015). Still, existing research suggests differences relating to sexual development in some people with ASD compared to typically developing (TD) people. In the current study, we focus on sexual orientation, gender identity and relationship status in adolescents and adults with ASD compared to peers in the general population.

First, findings relating to sexual orientation (feeling attraction to someone of the same sex, the other sex or both sexes) in adolescents and adults with ASD varied. Some studies found similar proportions of same sex attraction or experience (5–10%) among participants with ASD and TD peers (Dewinter et al. 2015; Fernandes et al. 2016), while other studies reported higher levels of non-heterosexual feelings and experiences in ASD (Barnett and Maticka-Tyndale 2015; Gilmour et al. 2012; Strunz et al. 2017) compared to the general population. In the US population (Copen et al. 2016) 17.4% of girls and women, and 6.2% of boys and men (age 18–44) reported same-sex experiences at some point during their lifetime. In this study 7.9% of men and 19% of women felt also or only attracted to someone of the same sex, and 7.7% of women and 4.9% men identified as other than heterosexual or straight. Prevalence data on sexual orientation in large groups of people with ASD are still lacking.

Second, a relationship between ASD and gender dysphoria (GD) was suggested (George and Stokes 2016; Glidden et al. 2016; van der Miesen et al. 2016). *Gender* refers to the behaviours and attitudes that, in a specific time and culture, are considered typical to males or females (Rosario and Schrimshaw 2014). While the number of people with GD is comparatively small, the prevalence of ASD characteristics in people referred to gender clinics is relatively high (de Vries et al. 2010; Pasterski et al. 2014; Skagerberg et al. 2015). Unfortunately, the findings regarding ASD and gender dysphoria are based on diverse samples (e.g. adolescents and adults with GD vs. participants with ASD) and not consistent across studies.

Different explanations were raised for this greater variation in sexual orientation and gender identity in people with ASD (George and Stokes 2016; Glidden et al. 2016; van der Miesen et al. 2016). Biological theories point to the influence of higher levels of antenatal testosterone exposure and to the assumption that GD and ASD share common genetic patterns. Psycho-social explanations include the effects of social experiences, the opportunity to meet people of the other sex, the role of sensory preferences, stereotyped interests and limited flexibility, and feeling less hampered by societal prejudices to come out. Empirical support for these mechanisms is mostly lacking. Yet, findings in the general population suggest that sexual minorities face several additional challenges (e.g. coming out) and risks (e.g. exclusion, abuse, violence) in their sexual development and well-being (de Graaf et al. 2015; Diamond 2013) compared to their peers. Little is known yet about the well-being and development of LGBT (i.e. Lesbian, Gay, Bisexual, Transgender) people with ASD (Bennett and Goodall 2016). Adolescents and adults with ASD and LGBT-feelings might face a double coming out: they might experience a sense of difference from the general population not only relating to their ASD-characteristics, but also pertaining to their gender identity, sexual orientation, or doubts about them. Better insight in sexual orientation and identity in adolescents and adults with ASD can lead to an increased awareness in sexuality education and support.

Third, research in samples of adults with ASD without cognitive impairments showed that 17% (Balfe and Tantam 2010) to 73% (Strunz et al. 2017) of people with ASD had a romantic relationship or were living with a partner. Being single was not negatively perceived by a third of the singles in the study of Strunz et al. (2017) and participants reported a variety of reasons why they did not feel up to being in a relationship (e.g. contact with a partner is exhausting). Singles with ASD can also experience sexuality in a positive way. However, some people with ASD without relationship experience compared to those with relationship experience showed poorer sexual functioning, e.g. less arousability, more anxiety or less desire (Byers et al. 2013). Still, results on relationship experience vary strongly and there is not yet a clear view of how this is experienced by adolescents and adults with ASD.

Most of the existing research is based on relatively small samples (the largest recent studies included n = 141 (Byers et al. 2012) and n = 229 (Strunz et al. 2017) participants with ASD) and case studies. Only the most recent studies are based on self-report. Recruitment of volunteers for sexuality related research might also have biased these results: volunteers in sexuality research were found to be more sexually experienced, to have a more positive affect towards sexuality and to be more interested in sexual novelty compared to controls in a non-sexual study (Bogaert 1996). In the current study, a large group of adolescents and adults with ASD reported on their sexual orientation, gender identity and relationship status. The findings on attraction and relationship status could be compared to those of peers in the general population. The results of this study can add to a nuanced and more realistic view on sexuality and relationships of people with ASD and offer input for sexuality education and support.

## Methods

### Participants

In 2016 675 adolescents (age >15) and adults with ASD registered in the Netherlands Autism Register (NAR) answered the questions for this study as a part of a broader survey. The NAR is a longitudinal register including over 2000 individuals with ASD. The NAR-participants registered themselves or their children voluntarily and completed a questionnaire on general demographic characteristics. Participants are invited annually to complete an additional online follow-up questionnaire.

Participants self-reported a diagnosis on the autism spectrum (see Table 1). Given the changes in DSM 5 (APA 2013), no differentiation between the different diagnostic categories was made in the analysis. The participant group consisted of a comparable number of men (48.3%) and women (51.7%) (see Table 1). This M:F ratio differs from the ratio found in the general ASD population, where ASD is more prevalent in men. The number of participants in the ASD group under the age of 25 is small (30 boys and 44 girls), which limits the statistical power in case of separate comparison of adolescents and adults.

**Table 1:** Sample characteristics

	Men		Women		Total	
	ASD	Controls	ASD	Controls	ASD	Controls
n (%)	326 (48.3)	3927 (48.7)	349 (51.7)	4137 (51.3)	675	8064
Age in years						
m (sd)	46.44 (14)	43.79 (15.9)	40.21 (12.4)	41.55 (15.9)	43.2 (13.5)	42.64 (15.9)
Range	15–80	15–70	16–75	15–70	15–80	15–70
Diagnosis: n (%)						
Autistic disorder	29 (8.9)	n/a	37 (10.6)	n/a	66 (9.8)	n/a
Asperger’s syndrome	167 (51)		173 (49.6)		340 (50.4)	
PDD-NOS	75 (23)		83 (23.8)		158 (23.4)	
MCDD	0 (0)		2 (0.6)		2 (0.3)	
ASS	54 (16.6)		51 (14.6)		105 (15.6)	
HFA	0 (0)		3 (0.86)		3 (0.4)	
No diagnosis	1 (0.3)		0 (0)		1 (0.15)	
Age ASD diagnosis						
m (sd)	37.12 (16.54)	n/a	32.92 (13.95)	n/a	34.96 (15.4)	n/a
Range	3.5–74		3.50–63.50		3.50–74	
AQ-28 score (cut-off 65)						
m (sd)	82.66 (12.3)	n/a	83.69 (10.9)	n/a	83.19 (11.6)	n/a
Range	50–110		48–110		48–110	
Education						
Primary	7 (2.1)	107 (2.6)	5 (1.4)	89 (2.2)	12 (1.8)	196 (2.4)
Vocational training	6 (1.8)	331 (8.2)	4 (1.1)	330 (8.2)	10 (1.5)	661 (8.2)
Special education	9 (2.8)	2 (0.05)	7 (2)	1 (0.02)	16 (2.4)	3 (0.04)
Lower secondary	50 (7.4)	557 (13.7)	24 (6.9)	708 (17.6)	50 (7.4)	1265 (15.7)
Higher secondary	78 (11.6)	463 (11.4)	44 (12.6)	515 (12.8)	78 (11.6)	978 (12.1)
Secondary vocational training	147 (21.8)	1216 (30)	80 (22.9)	1136 (28.3)	147 (21.8)	2352 (29.2)
Professional bachelor	199 (29.5)	1033 (25.5)	98 (28.1)	924 (23)	199 (29.5)	1957 (24.3)
University	124 (18.4)	332 (8.2)	63 (18.1)	291 (7.3)	124 (18.4)	623 (7.7)
Other	12 (4.7)	n/a	20 (5.7)	n/a	32 (4.7)	n/a
Unknown	7 (1)	11 (0.3)	4 (1.1)	18 (0.45)	7 (1)	29 (0.04)
Living situation^a^						
Living with parents	55	n/a	48	n/a	103	
Living independent	118		109		227	
Living independent with professional support	37		50		87	
Living together with partner and/or kids	111		112		233	
Group home	12		27		39	
Other	8		6		14	
Children						
Yes/no	140		128		301	
m (sd)	2.29 (1.1)		1.95 (0.9)		2.13 (1.02)	
Range	1–7		1–5		1–7	

^a^Some participants indicated more then 1 option, e.g. because they combine different living situations

### Controls

The questions used for this study were taken from the populations study *Sexual Health in the Netherlands 2011* (de Graaf 2012), allowing comparison with a large general population control group (n = 8064). The control and ASD group did not differ pertaining to the M:F ratio (***χ*** ^***2***^(1) = 0.40, n.s.) and to age (t(838.09) = −1.05, n.s.). The proportion of controls with a lower educational level (at highest completed lower secondary education) was significant, but in size only a little higher compared to the ASD group (*χ* ^*2*^(1) = 19.73, p < .01, Cramer’s *V* = 0.05).

### Measures

Nine questions were asked to explore the assigned gender at birth, gender identity, sexual orientation (sexual attraction and sex of the partner), relationship status and evaluation of relationship status, duration of the relationship, living situation, and whether the partner has (or is suspected of having) ASD. Sexual orientation was assessed using five response categories (1 = only men, 2 = mostly men, also women, 3 = equally to men and women, 4 = mostly women, also men, 5 = only women). The data on sexual attraction to both sexes (i.e. categories 2, 3 and 4) were combined in one category (feeling attraction to both sexes). The response categories for the question pertaining to gender identity and relationship status can be found in the Tables. These questions, except the question on gender identity, were part of the Sexual Health questionnaire (de Graaf 2012), enabling comparison of the ASD and control group answers. The general population group completed the whole sexual health questionnaire. In the ASD sample, only these nine questions were added to the NAR survey on functioning in different aspects of life.

The Autism Spectrum Quotient—Short Version (AQ-28; Hoekstra et al. 2011) is a 28-item questionnaire to assess autistic traits, including social skills, attention switching, a preference for routines, imagination and a fascination with numbers and patterns. The AQ-28 shows good sensitivity and specificity and has been shown to correlate highly with the original 50 item version of the measure (Hoekstra et al. 2011). The AQ-28 was included to describe and compare ASD characteristics of the participants in the ASD group.

### Procedure

The Medical Ethical Committee of the Vrije Universiteit Amsterdam approved this research. Participants registered in the NAR through the website (https://www.nederlandsautismeregister.nl). They received a link to complete a questionnaire on demographic characteristics, autism features (AQ-28: Hoekstra et al. 2011), diagnosis, physical condition and general well-being, sensory stimulus processing, housing, education, work or day care, leisure and special interests, partner relationship and social contacts, advocacy and research. Participants could decide to refuse participation at each invitation and decline further participation. Identification information was stored apart from all other data. The database with identification data and contact information is stored in separate, encrypted locations. All data used for this study were anonymised to the authors.

### Analysis

The participants belonged to a convenience sample, so no prior power calculations were performed. Data of men and women were separately analysed, since sex differences pertaining to sexual orientation, gender identity and romantic relationships are known to exist in the general population. Both groups were matched on age, and because of the small number of adolescents (age <25) no separate comparison of adults and adolescents were conducted. Chi square (*χ* ^2^) tests were used to test for differences in proportions comparing categorical variables. To detect medium to large effects based on Chi square analysis, a sample size of n = 88 would have been necessary. Effect size Cramer’s *V* is reported in case of a significant difference. Cohen (1988) suggested interpreting *V* = 0.10 as small, 0.30 as medium ,and 0.50 as large effect sizes. Independent *t*-tests were applied to compare means in continuous variables (age, AQ-scores). Effect size *r* will be reported, with the same benchmarks as described for *χ* ^2^ (*r* = .10 tot 0.50).

## Results

### Gender Identity

Most men and women in the ASD group identified conforming their assigned gender at birth (see Table 2). With both men and women, less than 1% identified as the opposite of their assigned gender at birth. About 22% of women and 8% of the men with ASD reported some gender non-conforming feelings. Data on gender identity were not available for the control group.

**Table 2:** Assigned gender at birth and gender identity

Assigned gender at birth	Male n (%)	Female n (%)
Feels male	299 (91.7)	3 (0.9)
Partly male, partly female	10 (3.1)	31 (8.9)
Not male, nor female	2 (0.6)	26 (7.4)
don’t know (yet)	4 (1.2)	9 (2.6)
Different (e.g. human, no sex)	8 (2.5)	8 (2.3)
Feels female	3 (0.9)	272 (77.9)

### Sexual Orientation

Sexual orientation was more varied in ASD compared to controls in both men (*Fisher’s exact* p < .*01, Cramer’s V* = *0.01*) and women (*χ* ^2^(3) = 339.68, p < .01, Cramer’s *V* = 0.27) (see Table 3). The proportion of participants with ASD that feels exclusively attracted to a partner of the opposite sex was smaller compared to the TD peers (men: *χ* ^2^(1) = 24.17, p < .01, *V* = 0.07, women: *χ* ^2^(1) = 227.25, p < .01, *V* = 0.22). Compared to the general population, more (about one in seven of the women and one in 20 men with ASD) indicated attraction other than to someone of the same and/or opposite sex (attraction towards men and/or women vs. other. Men: Fisher’s exact p < .01, Cramer’s *V* = 0.08, women: *χ* ^2^(1) = 222.06, p < .01, Cramer’s *V* = 0.22).

**Table 3:** Sexual attraction and relationship status

n (%)	Men		Women	
	ASD (n = 316)	TD (n = 3927)	ASD (n = 343)	TD (n = 4137)
Feels attracted to				
Men only	16 (5.1)	150 (3.8)	194 (56.6)**^a^	3601 (87)
Both men and women	27 (8.5)	184 (4.7)	77 (22.4)	418 (10.1)
Women only	258 (81.6)**^a^	3549 (90.4)	21 (6.1)	53 (1.3)
None of these	15 (4.7)	44 (1.1)	51 (14.9)	65 (1.6)
In a relationship	158 (50)**	2916 (74.3)	162 (47.2)**	2923 (70.7)
With a man	8 (5.1)	113 (3.9)	151 (93.2)	2861 (97.9)
With a woman	150 (94.9)	2803 (96.1)	11 (6.8)*	62 (2.1)
Living together with partner	136 (86.1)	2450 (84)	130 (80.2)	2324 (79.5)
Partner				
Diagnosed with ASD	12	n/a	25	n/a
Suspicion of ASD	7		29	

*p = .01, **p < .01

^a^Only attracted to the opposite sex versus other

More women with ASD were in a relationship with a woman compared to women in the general population, although the effect size of this finding was small (*χ* ^2^(1) = 14.48, *p* = .01, Cramer’s *V* = 0.07). The number of same-sex relationships in males did not differentiate between the ASD and control group (*χ* ^2^(1) = 0.56, n.s.).

Explorative comparison revealed that fewer adolescent boys (age <25) with ASD (63%) compared to adults with ASD (83%) reported only attraction to women (*χ* ^2^(1) = 10.11, p < .01, *V* = 0.176). In girls (56.8%) and women (55.4%) with ASD who reported attraction to men only, no significant difference appeared (*χ* ^2^(1) = 0.031, p > .05).

### Relationship Status

Fewer participants with ASD were in a relationship (50%) compared to controls (70%) (women: *χ* ^2^(1) = 81.06, p < .01, Cramer’s *V* = 0.13, men: *χ* ^2^(1) = 86.20, p < .01, Cramer’s *V* = 0.14), but among those who were in a relationship, comparable proportions of people with ASD and controls lived with their partner (women: *χ* ^2^(1) = 0.052, n.s., men: (*χ* ^2^(1) = 0.47, n.s.).

Among the participants with ASD, most relationships lasted longer than a year (men n = 150, women n = 156). Of the singles, 29% regretted their relationship status. Among those in a relationship, 9% felt dissatisfied, while 74% felt (very) satisfied. Men and women in a relationship were on average older than the singles (men: *M*_*rel*_ = 53, *sd* = 10.3, *M*_*single*_ = 41.5, *sd* = 14.5, *t*(283,65) = 8.11, p < .01, women: *M*_*rel*_ = 42.7, *sd* = 11.5, *M*_*single*_ = 39.2, *sd* = 13 *t*(340.99) = 2.70, p < .01). No difference in the level of autistic traits (AQ-28) emerged between singles and those in a relationship (independent *t*(614) = 1.30, p = .19).

## Discussion

This study shows that most people with and without ASD identify in line with their assigned gender at birth, feel attraction to someone of the other sex and have been in a heterosexual romantic relationship. However, adolescents and adults with ASD reported non-heterosexual attraction more often than their peers in the general population. Compared to Kuyper and Wijsen (2014), who found that 5.7% of men and 4% of women in a Dutch population study experienced an ambivalent or incongruent gender identity compared to their gender assigned at birth, the numbers in the present study, especially in women, appear high. However, this study allowed no statistical comparison with data pertaining to gender identity from the general population. Findings from earlier research on sexual diversity in adolescents with ASD compared to general population peers are inconsistent (Dewinter et al. 2015; Hellemans et al. 2007), though studies in adult participants confirmed a higher prevalence of non-heterosexual attraction (Gilmour et al. 2012; Strunz et al. 2017). The age (adults compared to adolescents) of the participants and the sample size in the present study might have made it possible to detect these differences pertaining to sexual orientation. In addition, a notable number of the men and women with ASD in this study indicated sexual attraction to neither men or women, possibly reflecting feelings of doubt, limited awareness of sexual orientation, or possibly the absence of sexual attraction. Some participants might have refused to answer questions pertaining to sexuality: ASD participants did not know in advance that questions about sexuality and attraction would be included in this survey, while the controls volunteered to participate in a study on sexual health. In addition, and in line with the conclusions of a review by Pecora et al. (2016), especially the women with ASD in our study reported same-sex attraction and did not strictly identify as female. The difference between men and women relating to sexual attraction is also found in the general population (e.g. Copen et al. 2016). Relating to gender identity, fewer women than men in the general population reported gender ambivalence or incongruence (Kuyper and Wijsen 2014) However, in this study, the variation in sexual attraction and gender identity seems even more pronounced in females with ASD compared to TD peers.

In general, little is known about sexual identity development in adults with ASD. Biological and psychosocial explanations have been proposed describing influences in different stages of development, such as prenatal hormone influences, feeling different in a neurotypical world, the interpretation of gender roles (what does it mean to feel male/female) or the need for concrete experience to decide about attraction. For now, a biopsychosocial view including all possible influences is defendable and probably more helpful than a single-factor explanation. Further research on the mechanisms behind identity and attraction development and ASD could clarify how to interpret these differences and how to support people.

About half of the participants with ASD in this study were in a relationship (in most cases a satisfying one), and most people with ASD who were in a relationship lived with their partner. This proportion is larger than in earlier studies (e.g. Balfe and Tantam 2010) and supports the recent findings of Strunz and colleagues (2017). The majority was in a relationship with an opposite-sex partner. In line with earlier findings (Byers et al. 2012), the people with ASD in a relationship were slightly older than the singles. No differences between men and women appeared concerning relationship experience, in contrast to earlier findings (Strunz et al. 2017). One in ten men and a third of the women with ASD reported having a partner with (or suspected of having) ASD, which was related to higher relationship satisfaction in the study of Strunz and colleagues (2017).

The current sample offers additional insight into sexuality in adults and adolescents with ASD because it was not recruited for sexuality related research, tackling potential response bias (Dunne et al. 1997; Strassberg and Lowe 1995). Possible bias related to differences in response style between autistic participants compared to those in the general population group cannot be excluded, e.g. autistic participants being less influenced by societal views or stereotypes on attraction and gender, or people who do not recognise themselves in the response categories. Some of these (unstudied) mechanisms might result in a more diverse picture of the autistic participants compared to the general population.

The participants declared that they have been diagnosed with ASD, which is also reflected in the high AQ scores. The overrepresentation of women in the group with ASD is remarkable and differed from the generally found M:F ratio of 4:1 (Levy et al. 2009), but is in line with general higher response rates of females compared to males in participating in research (Byers and Nichols 2014). Results from men and women were analysed separately and offered insight into sexuality in both sexes. However, several limitations should be considered when interpreting these findings. First, men and women with ASD in this study had a relative high education level and a late age of first diagnosis. Second, these findings are based on a limited number of single-item questions (Mustanski et al. 2014). The questions were embedded in different surveys for both groups and we do not know whether this influences the perception of and response to the questions. Third, statistical comparison of the data on gender identity in the ASD and general population group was not possible. Replication of these findings, and more in-depth research into the mechanisms behind identity development, relationship development, challenges in romantic relationships and into the needs of LGBT adolescents and adults with ASD can result in better support (Bennett and Goodall 2016). Attention to sexual and relationship diversity in autistic adolescents and adults, their needs and those of their relatives is needed in future research.

These findings have implications for clinical and educational practice. First, this study demonstrates that a large percentage of adolescents and adults with ASD have romantic experience and feelings. However, a larger group of people with ASD compared to typical peers remains single and longs for a relationship. Attention to their experience and needs is advised. Second, more adolescents and adults, especially women, report same-sex attraction and identify not completely or exclusively to their birth-assigned gender. This small group of men and women with ASD might face additional challenges (e.g. coming out) and risks (e.g. exclusion). Caregivers and professionals should be aware of and open towards these feelings and actively offer support when needed. In sexuality education and communication with adolescents with ASD, relationship skills, sexual diversity, and sexual identity development should be given appropriate attention.
